# Remote ischaemic preconditioning versus sham procedure for abdominal aortic aneurysm repair: an external feasibility randomized controlled trial

**DOI:** 10.1186/s13063-015-0899-3

**Published:** 2015-08-25

**Authors:** Ronelle Mouton, Jon Pollock, Jasmeet Soar, David C. Mitchell, Chris A. Rogers

**Affiliations:** Department Anaesthesia, Southmead Hospital, Bristol, BS10 5NB UK; Faculty of Health & Life Sciences, University of the West of England, Glenside Campus, Blackberry Hill, Bristol, BS16 1DD UK; Department Vascular Surgery, Southmead Hospital, Bristol, BS10 5NB UK; Clinical Trials and Evaluation Unit, School of Clinical Sciences, University of Bristol, Level 7, Bristol Royal Infirmary, Bristol, BS2 8HW UK

**Keywords:** Remote ischaemic preconditioning, Feasibility randomised controlled trial, Abdominal aortic aneurysm repair

## Abstract

**Background:**

Despite advances in perioperative care, elective abdominal aorta aneurysm (AAA) repair carries significant morbidity and mortality. Remote ischaemic preconditioning (RIC) is a physiological phenomenon whereby a brief episode of ischaemia-reperfusion protects against a subsequent longer ischaemic insult. Trials in cardiovascular surgery have shown that RIC can protect patients’ organs during surgery. The aim of this study was to investigate whether RIC could be successfully introduced in elective AAA repair and to obtain the information needed to design a multi-centre RCT.

**Methods:**

Consecutive patients presenting for elective AAA repair, using an endovascular (EVAR) or open procedure, in a single large city hospital in the UK were assessed for trial eligibility. Patients who consented to participate were randomized to receive RIC (three cycles of 5 min ischaemia followed by 5 min reperfusion in the upper arm immediately before surgery) or a sham procedure. Patients were followed up for 6 months. We assessed eligibility and consent rates, the logistics of RIC implementation, randomization, blinding, data capture, patient and staff opinion, and variability and frequency of clinical outcome measures.

**Results:**

Between January 2010 and December 2012, 98 patients were referred for AAA repair, 93 were screened, 85 (91 %) were eligible, 70 were approached for participation and 69 consented to participate; 34 were randomized to RIC and 35 to the sham procedure. There was a greater than expected variation in the complexity of EVAR that impacted the outcomes. Acute kidney injury occurred in 28 (AKIN 1: 23 %; AKIN 2: 15 % and AKIN 3: 3 %) and 7 (10 %) had a perioperative myocardial infarction. Blinding was successful, and interviews with participants and staff indicated that the procedure was acceptable. There were no adverse events secondary to the intervention in the 6 months following the intervention.

**Conclusions:**

This study provided essential information for the planning and design of a multi-centre RCT to assess effectiveness of RIC for improving clinical outcomes in elective AAA repair. Patient consent was high, and the RIC intervention was carried out with minimal disruption to clinical care. The allocation scheme for a definite trial should take into account both the surgical procedure and its complexity to avoid confounding the effect of the RIC, as was observed in this study.

**Trial registration:**

Current Controlled Trials ISRCTN19332276 (date of registration: 16 March 2012). The trial protocol is available from the corresponding author.

**Electronic supplementary material:**

The online version of this article (doi:10.1186/s13063-015-0899-3) contains supplementary material, which is available to authorized users.

## Background

The prevalence of abdominal aortic aneurysm (AAA) in men aged 65 to 74 years is 5 to 7 %, and increases to over 10 % in those over the age of 74 years [1]. Patients undergoing elective AAA repair are typically a high-risk and vulnerable group with comorbidities [2–4]. They are at risk of sustaining perioperative myocardial injury (11-22 %), renal impairment (4.2- 20 %) and stroke (1 to 2 %) [3, 5, 6]. Perioperative cardiac injury is the most common cause of morbidity and mortality after AAA repair, and it is associated with poor short and long-term clinical outcomes [7–9]. Up to 70 % of patients undergoing AAA surgery have significant coronary artery disease, but a large randomised trial failed to demonstrate any significant benefit from prophylactic coronary revascularisation [10, 11]. Hence there is a need to find new treatment strategies to reduce the risk of complications following AAA surgery.

Remote ischaemic preconditioning (RIC) is a phenomenon in which a brief period of imposed sub-lethal ischaemia followed by reperfusion in one area of the body can provide systemic protection against cellular injury during a subsequent prolonged ischaemic episode [12]. Since the concept of RIC was first described, numerous laboratory studies have shown that RIC elicited through *transient limb ischaemia* can reduce ischaemia-reperfusion injury by a preconditioning signal. The mechanisms involve mediators (for example, adenosine, bradykinin and endogenous opioids) that are generated during ischaemia, a cascade of second messengers and target organ effectors. RIC also activates the autonomic nervous system and humoral mediators to ensure systemic spread of protection [13, 14].

Evidence for the clinical effectiveness of RIC is mixed. Several small and one larger randomised controlled trial (RCT) (n = 329) have shown that RIC is protective in patients with acute myocardial infarction (MI) and in those undergoing cardiovascular surgery or percutaneous coronary intervention [15–18]. Significant decreases in troponin-T release were observed, and the larger trial in cardiac surgery patients showed a reduced risk of MI and all-cause mortality [18]. A systematic review in cardiac surgery demonstrated a significant benefit of RIC over controls in reducing the biomarkers of cardiac injury [19], but a meta-analysis could not demonstrate any advantage of RIC in preventing acute kidney injury after cardiovascular interventions [20]. In vascular surgery, a Cochrane review identified four small RCTs (n = 232) evaluating RIC, and no overall beneficial effect was demonstrated [21].

Vascular surgery has undergone significant change in recent years, with an increasing use of endovascular (EVAR) interventions instead of open surgery. In the UK there is also movement towards the centralisation of vascular surgery, and the AAA Quality Improvement Programme supports more emphasis on patient-centred care and an improved patient pathway [22, 23]. In the context of these changes, as patients can have either an endovascular or an open surgical AAA repair, it was not clear that a large, adequately-powered, multi-centre RCT of RIC versus sham, with blinded care providers and outcome assessors, was possible. We conducted an external pilot study to determine whether a trial was feasible and to inform the choice of primary outcome for a future large multi-centre RCT. Here, we report the findings of this feasibility study, which was funded by the UK National Institute for Health Research (NIHR) Research for Patient Benefit programme (PB-PG-0609-18150).

## Methods

### Study design

This study was designed as a blinded, single-centre, parallel group feasibility RCT with a 1:1 allocation ratio (see Additional file 1 for CONSORT checklist).

### Participants

Patients referred for a primary elective AAA repair as an EVAR or open procedure were eligible to take part. Patients taking sulphonylurea oral hypoglycaemic drugs or nicorandil, which are known to influence preconditioning, were excluded [24, 25].

### Study setting

The study was conducted at a regional vascular surgery centre in Bristol, United Kingdom. The study was approved by the South West Frenchay Research Ethics Committee (ref. 10/H0107/36) and sponsored by the North Bristol NHS Trust. Patients were informed about the study and given a study information sheet when they attended the consultant-led preoperative assessment clinic 1 to 3 weeks before surgery. Written informed consent was obtained the day before surgery from patients willing to participate, when they attended the hospital for the operation.

### Interventions

Participants were randomised to either the RIC or sham procedure. The RIC intervention consisted of three 10-min cycles of 5 min of arm ischaemia (inflation of a blood pressure cuff to 40 mmHg above the patient’s systolic blood pressure) followed by 5 min of reperfusion (30 min in total), immediately before surgery. The sham procedure consisted of a pressure cuff inflated for the same periods as the RIC intervention but only to 40 mmHg. Both interventions were carried out by the anaesthetic team as part of the perioperative care.

Standard surgical and anaesthetic techniques were used in both groups. All participants had an arterial cannula inserted for continuous blood pressure monitoring, and a urinary catheter inserted after induction of anaesthesia and before surgery commenced. Participants who underwent open surgery also had a thoracic epidural and a central venous catheter inserted before surgery commenced. Active warming with fluid warmers and warming blankets were used for every case. Participants were extubated at the end of surgery and transferred to a high dependency area for ongoing care.

### Outcomes

The study outcomes were as follows:

1. Number of patients referred for elective AAA repair and the number and proportion eligible for the trial.
2. Number of eligible patients approached to participate and reasons for non-approach.
3. Number and proportion of eligible patients approached who consented to participate and reasons for non-consent.
4. Success of blinding those not involved directly in administering the intervention.
5. Impact of the intervention on delivery of care and time taken for anaesthesia (defined as the time from when the patient entered the theatre suite until surgical knife to skin).
6. Participant follow-up to 6 months, numbers and reasons for loss to follow-up
7. Completeness of clinical outcome data and frequency of the these events, in particular, indices of acute renal injury within the first 48 hours after AAA repair, as defined by the AKIN criteria [26]; cardiac events, namely myocardial infarction (MI), new arrhythmias, electrocardiogram (ECG) changes and raised troponin T levels in the period prior to discharge; and complications, including stroke and death, up to 6 months after surgery.
8. Staff and participant views on the intervention and study conduct.

### Sample size

A sample size of 60 participants, 30 Open and 30 EVAR procedures, was chosen to provide recruitment estimates with sufficient precision to inform a larger definitive trial; for example 50 % eligible patients recruited (worse-case scenario) would be estimated with a 95 % confidence interval of ± 13 %. The study was not powered to compare outcomes between the RIC and sham groups. A study of 60 participants is also in line with recommendations for pilot/feasibility studies [27]. An independent Data Monitoring and Safety Committee periodically reviewed the safety data.

### Randomisation

The randomisation sequence, with a 1:1 allocation, varying block sizes and stratified by type of surgery (OPEN or EVAR), was generated by computer. Randomisation took place after written informed consent had been obtained. Allocations were accessed via a secure password-protected website and were concealed until sufficient information to uniquely identify the individual had been entered. Randomisation was carried out by a member of the anaesthetic team on the morning of surgery.

### Blinding

With the exception of the in-theatre anaesthetic team who administered the intervention, everyone (participants, surgeons, nursing staff and research nurses) was blinded to the intervention received.

### Data and sample collection

Blood samples to measure serum creatinine and troponin T were taken preoperatively, immediately after surgery, and on the morning of the first two postoperative days as part of routine care. Urine output was measured for the first 48 hours. Research nurses were responsible for collecting all study data, with the exception of data on the timing of the intervention and inflation pressures used, and on anaesthetic and procedure times. These latter data were collected by the anaesthetist administering the intervention. Participants were interviewed by a study research nurse before hospital discharge and were contacted again at 6 months by telephone. Their views on the trial and the treatment received (RIC or sham) were sought, and at 6 months, details of any complications following discharge after surgery were collected. Staff interviews were conducted by a research nurse after all clinical staff had some experience of the trial. Responses to nine interview questions were recorded qualitatively and then coded. The final analysis was carried out by an experienced qualitative researcher (JP). Specific questions for patient and staff interviews are attached as Additional file 2.

### Patient-public involvement

Patients provided input during all stages of the project. Those consulted included 18 patients who had previously undergone AAA surgery; two patients who had cardiac or renal transplant surgery; and 12 members of the Avon, Gloucestershire, Wiltshire and Somerset (AGWS) Cardiac and Stroke Network Patient, Carer and Public Involvement Group. Before the start of the study, the project was presented to this latter group. Feedback was provided anonymously. Patients who had previous AAA surgery and expressed an interest to take part in the research, reviewed and commented on the study information sheets and recruitment process. Two patient representatives were members of the steering committee and reviewed all progress reports.

### Statistical analysis

Continuous variables were summarised using the mean and standard deviation, and categorical data were summarised as a number and percentage. Participants were grouped by treatment allocation (intention to treat).

## Results

### Patient recruitment

In total, 98 patients were referred for an elective AAA repair (71 EVAR and 27 open procedures) during the 2-year study period from 1 January 2011 to 31 December 2012. Of these, 93 were screened for inclusion and 85 (91 %) were eligible to take part. Fifteen eligible patients were not approached for the trial:

1. Three because the surgery was cancelled (patients with significant co-morbidities where consultation at the multi-disciplinary team meeting and patient choice led to the decision not to proceed).
2. Six for logistical reasons (staff shortage; initially not enough backup when study principal investigator (PI) was absent).
3. Six because the trial in patients undergoing an EVAR procedure was temporarily suspended while the results of an interim analysis was reviewed.

**Fig. 1:**
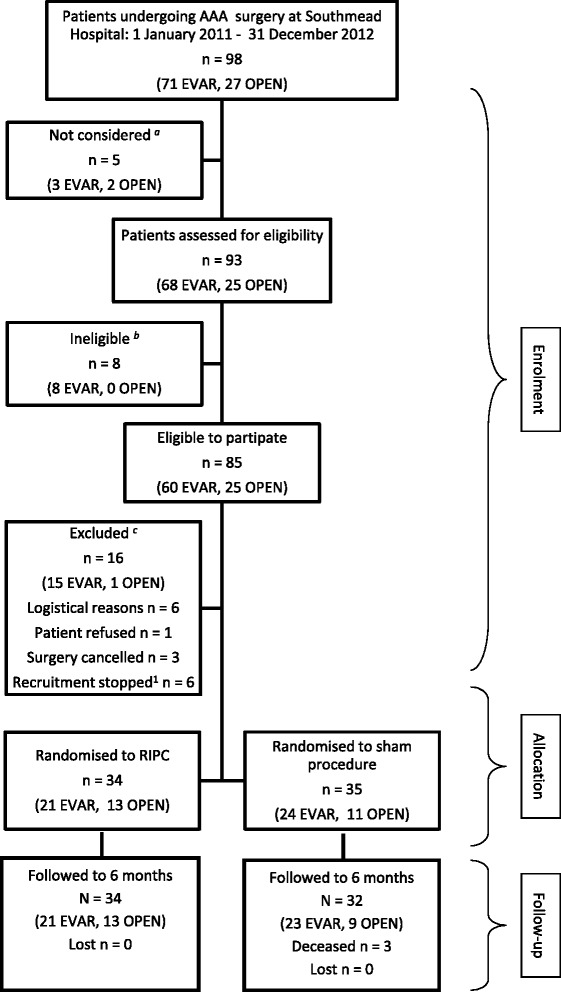
Study flow diagram. ^*a*^Failure to identify patients presenting for elective AAA surgery early enough at the start of study and therefore unable to supply study information to patients in appropriate time scale. ^*b*^Patient on sulphonylurea type drugs or nicorandil (6); Lacked capacity (2). ^*c*^Unavailability and shortage of research staff to facilitate recruitment (6); Surgery cancelled (3); Patient refused (1); recruitment of patients undergoing EVAR was temporarily suspended while the results of the interim analysis were reviewed (6)

The remaining 70 patients were invited to join the trial and 69 (99 % of those invited, 81 % of those eligible) patients consented to participate. The one person who declined did so because he felt overwhelmed and did not want to be burdened with any additional information. Thirty-four participants were randomised to RIC, and 35, to the sham procedure (Fig. 1).

### Protocol violations

There was one protocol violation. One participant undergoing an EVAR was randomised to RIC but did not receive the intervention. This participant had significant co-morbidities; intra-operative clinical need escalated, took preference and the consultant anaesthetist decided not to complete the RIC intervention.

### Baseline characteristics

Patient characteristics at baseline are presented in Table 1. The mean age was 72 years. The groups were well matched for age, anaerobic threshold, VO**2** max, Physiological and Operative Severity Score for the enUmeration of Mortality and Morbidity (VPOSSUM) score, the presence of hypertension and cerebrovascular disease. The baseline renal function and haematological values were also similar. Proportionally, more participants in the RIC group had a history of ischaemic heart disease (38 % versus 26 %) and/or congestive cardiac failure (15 % versus 3 %). The number of EVAR procedures was similar in each group (21 in the RIC group versus 24 in the sham group), but by chance, the number of complex EVAR procedures (participants with pre-significant anatomical difficulties requiring longer and extra procedures identified preoperatively) was higher in the RIC group (10/21 versus 1/24). Baseline patient characteristics by operative procedure are attached as Additional file 3.

**Table 1:** Baseline patient characteristics

	Randomised allocation	
	RIC (n = 34)	SHAM (n = 35)
Age (years)	72 (6)	72 (7)
Creatinine (μmol/L)	102 (52)	91 (19)
Urea (mmol/L)	7 (4)	6 (2)
Hb (g/dL)	14 (2)	14 (2)
Albumin (g/L)	37 (5)	37 (4)
Anaerobic threshold (ml/kg/min)	12 (2)	13 (2)
VO_2_ max (ml/kg/min)	14 (3)	17 (6)
VE/VCO_2_ (l/l)	33 (6)	31 (4)
V-POSSUM	19 (4)	19 (4)
ACEI	20 (59 %)	17 (49 %)
Statin	26 (77 %)	25 (71 %)
Beta-blocker	12 (35 %)	11 (31 %)
Hypertension	26 (77 %)	25 (71 %)
Ischaemic heart disease	13 (38 %)	9 (26 %)
Cerebrovascular disease	6 (18 %)	7 (20 %)
Congestive cardiac failure	5 (15 %)	1 (3 %)
Predicted complex EVAR	10 (29 %)	1 (3 %)

Data are shown as mean (SD) or n (%) as appropriate, unless indicated otherwise

VO_2_ Max is the maximal oxygen uptake in 1 min during maximal exhaustive exercise

VE/VCO_2_ is the ventilatory equivalent for carbon dioxide (an indicator of ventilatory efficiency)

V-POSSUM stands for the risk profile measured by the Physiological and Operative Severity Score for the enUmeration of Mortality and Morbidity

ACEI = angiotensin converting enzyme inhibitor

### Operative details

The high number of more complex EVAR procedures in the RIC group was reflected in the procedure times. The mean surgical procedure time (defined as the time from knife to skin until dressings on after closure) for EVAR in the RIC group was 2.96 hours compared to a mean of 2.28 hours in the sham group. In contrast, for open surgery, the mean surgical procedure time was on average 48 min shorter in the RIC group. Anaesthetic times (defined as the time from patient entry to the anaesthetic room until start of surgery) showed a similar pattern to the procedure times (Table 2).

**Table 2:** Operative characteristics

	Randomised allocation	
	RIC (n = 34)	SHAM (n = 35)
Procedure time (hours)		
EVAR	2.96 (0.86)	2.28 (0.45)
Open	4.07 (0.22)	4.87 (0.73)
Anaesthetic time (hours)		
EVAR	0.71 (0.05)	0.64 (0.04)
Open	1.16 (0.10)	1.36 (0.23)

Data are shown as mean (SD)

### Intervention

There were no adverse outcomes such as petechiae, numbness, tingling or nerve injuries reported secondary to the RIC intervention.

### Post-operative complications

Overall, 28/69 participants (41 %) had a degree of acute kidney injury following surgery. In the majority of cases the injury was graded as AKIN 1 (16 participants, 23 %, Table 3) Acute kidney injury occurred more frequently in the RIC group (47 % versus 34 %). Cardiac events were also more common in the RIC group (MI 15 % versus 6 %; new arrhythmias 21 % versus 14 %; Troponin T > 14 ng/L 47 % versus 29), there were three deaths, all in the sham group; two participants had open surgery; and one an EVAR procedure. Clinical outcome data was complete for all participants, with the exception in one participant in the sham group in whom troponin T was not measured. Patient outcome data by operative procedure are included in Additional file 3.

**Table 3:** Clinical Outcomes

	Randomised allocation	
	RIC (n = 34)	SHAM (n = 35)
Acute kidney injury		
AKIN 1	9 (27 %)	7 (20 %)
AKIN 2	7 (21 %)	3 (9 %)
AKIN 3	0 (0 %)	2 (6 %)
Myocardial Infarction	5 (15 %)	2 (6 %)
New post-op ECG changes	7(20 %)	7 (20 %)
New arrhythmia	7 (21 %)	5 (14 %)
Troponin T > 14 ng/L	16 (47 %)	10 (29 %)
Other post-operative issues^a^	13 (38 %)	12 (34 %)
Death	0 (0 %)	3 (9 %)

Data are shown as n (%) and cover the period from surgery to 6 months

^a^Wound infection, buttock or groin pain, pyrexia of unknown origin, graft problems

AKIN refers to Acute Kidney Injury as classified by Acute Kidney Injury Network [26]

### Clinical outcomes after hospital discharge to 6 months

All 66 surviving participants were followed-up to six months. Fifty (76 %) of the 66 participants indicated that they had no new health complaints since the surgery. The remainder reported complications related to existing co-morbidities (gradual worsening in Parkinson’s disease, dementia, and depression); or the surgery (six patients reported buttock pain and one wound breakdown).

### Interim analysis

Interim analyses, carried out by a statistician independent of the study team, were planned after 20, 40 and 60 participants had been recruited. No concerns were raised after the first two analyses, but the third analysis identified a greater frequency of renal and cardiac complications in participants undergoing EVAR in the RIC group. As a result, recruitment of patients having an EVAR procedure was temporarily halted while the data were investigated further. This investigation revealed that the increased complication rate was not related to the intervention but to the predicted complexity of the EVAR procedure. The patients who had more complex EVAR surgery stayed in the hospital longer and had more complications. The findings were discussed by the study steering committee and it was agreed that recruitment should be restarted.

### Participant and staff interviews

All surviving participants interviewed at 6 months found the intervention acceptable and were supportive of the research. None expressed knowledge of allocation. In total, 20 staff interviews were conducted. The staff interviews covered questions about blinding and the impact of RIC on staff workload and activities. Staff interviews were conducted between August 2011 and April 2013 after the staff all had some experience of the trial. No staff member not involved in the intervention activity itself expressed knowledge of allocation. Of the 20 staff interviewed, nine (45 %) were part of the anaesthetic team responsible for delivering the intervention and 11 (55 %) were members of the theatre team, including four surgeons, one radiologist, and six scrub and theatre staff. From the staff interview questions, 86 % indicated no or minimal effect of the intervention on day-to-day practice, 11 % reported a small effect or minor point, and 1 % a moderate effect or significant point. The surgeons and radiologist all universally rated the intervention as having minimal impact on normal work patterns.

### Patient-public involvement

The patient, carer and public involvement group of the AGWS considered the study important for patients. They advised on how best to integrate the trial into the patient pathway and recommended that when the trial was discussed with potential participants during the preoperative assessment the patient’s blood pressure be taken to illustrate the RIC process. Patients who had previous AAA surgery suggested changes to the patient information leaflet and gave feedback on how and by whom patients prefer to be approached for research. The two patient representatives on the Trial Steering Committee wish to continue to be involved in further proposals based on this feasibility study.

## Discussion

This study shows that an RCT to study the RIC intervention in patients undergoing elective AAA repair is feasible to conduct and acceptable to patients and the clinical team. This study also provides essential information for the planning and structuring of an adequately powered multi-centre RCT to assess whether the RIC intervention can provide any benefit to patients who require elective AAA repair.

Surgery for AAA repair is undergoing a period of significant change both globally and in the UK [22, 28]. We had intended to recruit 30 participants having an EVAR and 30 having open surgery. This was not achieved because a significantly greater proportion of patients (72 %) were referred for an EVAR procedure than predicted when the study was conceived. We could have chosen to stop recruitment to EVAR once our target of 30 participants was reached, but we decided against this for the following reasons: first, it would have prevented us obtaining recruitment estimates across the two surgical procedures, which reflects current clinical practice, and secondly we wanted to maintain study momentum and recruitment procedures and not disrupt the logistics of the study. We sought and obtained ethical committee approval to continue recruitment of patients presenting for EVAR surgery beyond our original target for this group. The response to RIC might differ between EVAR and open surgery, but this is not something we can evaluate in this study as it was not powered for comparative analysis. Any future trial should include patients from both the open and EVAR groups and be adequately powered to evaluate the effects of RIC on each type of AAA repair in a subgroup analysis.

There was variation in the complexity of EVAR surgery that might have impacted the clinical outcomes measured in this study. Across the trial, there were proportionally more AKI and cardiac events in the group of 11 complex EVAR procedures (6/11 versus 22/57 AKI events and 3/11 versus 4/58 MI). In all cases the anatomical complexities were identified before the procedure commenced. It has been shown that a high anatomic severity grading score correlates with an increase in surgical operating time, length of hospital stay, blood loss, contrast use and cost [29]. Randomization was stratified by type of procedure but did not include stratification or minimization by surgical complexity. By chance, as can happen, particularly when sample sizes are small, a greater number of participants requiring a more complex and longer EVAR procedure were randomized to RIC compared to sham. In participants who had a predicted complex EVAR, the procedure took on average 50 min longer. The randomization scheme for an adequately powered definite RCT should take into account surgical procedure, surgical complexity and participant risk, to avoid the imbalance between groups in these potential confounders, as was observed in this study.

The incidence of both acute kidney injury and cardiac events after elective AAA surgery were high (Table 3), which is in line with previous reports in the literature [3, 4] and consistent with a recently published study, where 28/62 (45 %) of patients suffered acute kidney injury after elective open AAA repair [30]. Pulmonary injury and intestinal injury are also common after elective open AAA repair; another small study (n = 62) has suggested that RIC might be protective against these complications in this population [31].

Based on this study, cardiac events or renal injury could be chosen as a primary outcome measure for a future trial; these complications have a high incidence, are important to patients, impact NHS resources and were measured reliably with minimal missing data. This study is the first to report on the acceptability of the RIC intervention to participants and staff. Feedback indicated that it can be incorporated into the day-today clinical setting without affecting anaesthetic or procedure times. No RIC-related safety issues were reported.

Patients undergoing elective AAA repair are a high risk and vulnerable group with many co-morbidities. The risk profile, measured by V-POSSUM score, for participants undergoing elective AAA surgery in this study was 19 on average, indicating a morbidity prediction of 44.8 % and a mortality prediction of 12.8 % for elective AAA surgery in our usual patient population [32]. Complications are common after both open and EVAR procedures and lead to prolonged periods in intensive care and delayed recovery, with negative effects for patients and significant costs to health services. The hospital cost per patient is estimated between £9,946 and £13,257 for patients undergoing elective AAA surgery [33]. Currently the demand for elective AAA repair is increasing and, due to the aging population, as well as the new UK national screening program for men over 65 years, it will continue to increase in the future [1, 2, 4]. The high cost of AAA repair coupled with the significant morbidity and mortality means that there is a need to identify interventions that can reduce harm and improve patient outcomes. RIC is of great interest because of its simplicity, safety, non-invasive nature and low cost. This makes the RIC intervention attractive for testing in a large pragmatic clinical trial. There have been other small, single-centre, clinical studies on the effect or RIC in patients who are undergoing elective AAA surgery [17, 21, 30, 31]. Large multi-centre RCTs, with blinded care providers and outcome assessors are now needed to investigate if RIC is safe and can benefit this patient group.

## Conclusions

Our study considered the feasibility of undertaking a RCT, and as such, it was not adequately powered to assess the effectiveness of the RIC intervention in the patient population studied. This study provided essential information for the planning and design of a multi-centre RCT to assess effectiveness of RIC for improving clinical outcomes in elective AAA repair. Our data suggest that a large multi-centre trial is feasible with modifications; the majority of patients were eligible, patient consent was high and the RIC intervention can be carried out with minimal disruption to clinical care, but there was imbalance in the surgical complexity between the groups, which impacted outcomes. A future trial would need to minimise the chance of such imbalance.

## Additional files

Additional file 1:
**CONSORT Checklist.** (DOC 217 kb) Additional file 2:
**Specific questions used by the interviewer for the patient and staff interviews.** (DOCX 17 kb) Additional file 3:
**3a Baseline patient characteristics by operative procedure.** Data are shown as mean (SD) or n (%) as appropriate, unless indicated otherwise. VO_2_ Max is the maximal oxygen uptake in 1 min during maximal exhaustive exercise. VE/VCO_2_ is the ventilatory equivalent for carbon dioxide (an indicator of ventilatory efficiency). V-POSSUM stands for the risk profile measured by the Physiological and Operative Severity Score for the enUmeration of Mortality and Morbidity. ACEI = angiotensin converting enzyme inhibitor. 3b: Outcome by operative procedure. Data are shown as n (%) and cover the period from surgery to 6 months. *Wound infection, buttock or groin pain, pyrexia of unknown origin, graft problems. AKIN refers to Acute Kidney Injury as classified by Acute Kidney Injury Network [26]. (DOCX 17 kb)

## Abbreviations

AAA: Abdominal aortic aneurysm
AGWS: Avon, Gloucestershire, Wiltshire and Somerset
ACEI: Ace inhibitor
AKI: Acute kidney injury
AKIN: Acute kidney injury network
ECG: Electrocardiogram
EVAR: Endovascular aneurysm repair
NIHR: National Institute for Health Research
NHS: National Health Service
MI: Myocardial infarction
RIC: Remote ischaemic preconditioning
RCT: Randomised controlled trial
SD: Standard deviation
UK: United Kingdom
VPOSSUM: Physiological and operative severity score for the enumeration of mortality and morbidity
