# Supplementary material for: Remote ischaemic preconditioning versus sham procedure for abdominal aortic aneurysm repair: an external feasibility randomized controlled trial
Source: Trials. 2015 Aug 25;16:377. doi: 10.1186/s13063-015-0899-3 (PMC4549128; doi:10.1186/s13063-015-0899-3)
Supplement: Additional file 2: — Specific questions used by the interviewer for the patient and staff interviews. (DOCX 17 kb) [file 13063_2015_899_MOESM2_ESM.docx]

**Health staff interview topic sheet** *(Version 1.2 )*

1. How would you describe the overall impact of the additional procedure on peri-operative care?
   *Please provide details of the impact(s).*
2. Did the additional procedure alter the timing of any of your duties during peri-operative care?
   *If so, how?*
3. Were any delays caused by the additional procedure?
   *If so, how and please provide details?*
4. Have you experienced or witnessed any difficulties experienced by staff in implementing or managing the additional procedure?
   *Please provide details of any difficulties*
5. Have there been any instances of needing to allocate any duties to other staff as a result of implementing the additional procedure?
   *Please can you give examples and which staff grade and duties were involved?*
6. Have you observed any interference in the course of peri-operative care caused by implementing the additional procedure?
   *Please give details of any interference you have observed or are aware of*
7. Are you aware of any opinions of your colleagues involved in peri-operative care about implementing the additional procedure?
   *Please list any opinions from any colleagues involved in the care of these patients*
8. How would you describe your views on incorporating this procedure in routine peri-operative care?
   *Do you have any positive or negative views about this and what are they?*
9. Is there anything else you might wish to add about your experience of implementing the procedure yourself or seeing how it affects the peri-operative care period?

**Patient interview topic sheet** *(Version 1.2 )*

1. ***Patients that do not give their consent to participate in the trial***

Interviewer: “Thank you for considering this request. It is fine if you do not wish to participate and this will not affect the care you receive in any way. …….

But because we need to know what ALL patients feel about joining a trial like this, whether they agree to or not, might I ask you a couple of questions about your feelings about being approached to join the trial? It will take about 5 minutes. Any comments you make would be completely anonymous, but, again, if you do not wish to answer any questions or express your views that is absolutely fine”

Patient response option 1 : “No, I do not want to talk any more about this”

Interviewer: “OK. Thanks very much for your time”

End of contact

Patient response option 2: “Alright, what do you want to know?”

Interviewer: “Well we just want to know what you feel about the trial overall and the request to participate….. for example:

Did you have any particular reasons for not joining the trial?
 *Probe details*

Did you have any fears or worries about being in the trial?
 *Probe details*

Was there anything about the way you were approached to participate that caused
 you concern?

*Probe details and suggestions for improvement*
 Was there anything in the information sheet that raised concerns or uncertainties
 about being in the trial?
 *Probe details and suggestions for improvement*

Do you understand what we are trying to find out in this trial?

*Probe understanding and any perceived understanding failure*

Is there anything else you would like to say about your feelings about participation
 in the trial?

*Probe details*

That is all. Many thanks for your time…..”

1. **Consenting patient exit interview topics**After introductions and a reminder of their participation in the trial…

   Interviewer: “ Thank you again for having participated in the trial. Because this is a feasibility study we need to know as much as possible about patients’ feelings about having been involved in the trial. Might I ask you a few questions about your participation in the trial? It will take about 10 minutes. Any comments you make would be completely anonymous, but if you do not wish to answer any questions or express your views that is absolutely fine, this will not affect the care you receive in any way”

Patient response option 1 : “No, I do not want to talk any more about this”

Interviewer: “OK. Thanks very much for your time”

End of contact

Patient response option 2: “Alright, what do you want to know?”

Interviewer: “Well we just want to know what you feel about the trial overall and your involvement in it….. for example:

Did you have any particular reasons for joining the trial?
 *Yes, No, Unsure*

*Probe details*

Did you have any fears or worries about being in the trial itself?
 *Yes, No, Not really*

*Probe details*

Was there anything about the way you were approached to participate that caused
 you concern?

*Yes, No, Not really*

*Probe details and suggestions for improvement*
 Was there anything in the information sheet that raised concerns or uncertainties
 about being in the trial?
 *Probe details and suggestions for improvement*

Did you receive enough information about the trial?

*Yes, No, Unsure
 Probe details and suggestions for improvement*

Were you able to ask questions and receive satisfactory answers?

*Yes, No, Unsure*

*Probe details and suggestions for improvement*

What do you feel about the process used for obtaining consent ?

*Very good, Alright, Neutral, Not good, Very poor*

*Probe details and suggestions for improvement*

Do you understand what we are trying to find out in this trial?

*Yes, No, Not really*

*Probe understanding and any perceived understanding failure*

Do you know what group you were in for this trial? The blood pressure group or the
 no intervention group?

*Intervention, Control*

*For either probe how strongly they believed their answer and what made them
 believe this?*

Since the operation have you experienced any pain or soreness in your arms
***By arm****: Yes, No, Not really
Probe type of pain, soreness, duration of symptoms*

Is there anything else you would like to say about your feelings about participation
 in the trial?

*Yes, No
Probe details*

That is all. Many thanks for your time…..”
