# Supplementary material for: Remote ischaemic preconditioning versus sham procedure for abdominal aortic aneurysm repair: an external feasibility randomized controlled trial
Source: Trials. 2015 Aug 25;16:377. doi: 10.1186/s13063-015-0899-3 (PMC4549128; doi:10.1186/s13063-015-0899-3)
Supplement: Additional file 3: — 3a Baseline patient characteristics by operative procedure. Data are shown as mean (SD) or n (%) as appropriate, unless indicated otherwise. VO2 Max is the maximal oxygen uptake in 1 min during maximal exhaustive exercise. VE/VCO2 is the ventilatory equivalent for carbon dioxide (an indicator of ventilatory efficiency). V-POSSUM stands for the risk profile measured by the Physiological and Operative Severity Score for the enUmeration of Mortality and Morbidity. ACEI = angiotensin converting enzyme inhibitor. 3b: Outcome by operative procedure. Data are shown as n (%) and cover the period from surgery to 6 months. *Wound infection, buttock or groin pain, pyrexia of unknown origin, graft problems. AKIN refers to Acute Kidney Injury as classified by Acute Kidney Injury Network [26]. (DOCX 17 kb) [file 13063_2015_899_MOESM3_ESM.docx]

**Additional File 3**

**3a. Baseline patient characteristics by operative procedure**

**Legend:**

Data are shown as mean (SD) or n (%) as appropriate, unless indicated otherwise.

VO_2_ Max is the maximal oxygen uptake in one minute during maximal exhaustive exercise

VE/VCO_2_ is the ventilatory equivalent for carbon dioxide (an indicator of ventilatory efficiency)

V-POSSUM stands for the risk profile measured by the Physiological and Operative Severity Score for the enUmeration of Mortality and Morbidity[29].

ACEI = angiotensin converting enzyme inhibitor

|  | **EVAR (n=45)** | | **OPEN (n=24)** | |
| --- | --- | --- | --- | --- |
|  | **Randomised allocation** | | **Randomised allocation** | |
|  | **RIC (n=21)** | **SHAM (n=24)** | **RIC (n=13)** | **SHAM (n=11)** |
| Age (years) | 73 (7) | 73 (7) | 72 (6) | 72 (8) |
| Creatinine (μmol/L) | 106 (63) | 93 (20) | 95 (25) | 87 (18) |
| Urea (mmol/L) | 7 (5) | 6 (2) | 6 (2) | 6 (1) |
| Hb (g/dL) | 14 (2) | 14 (2) | 14 (1) | 14 (2) |
| Albumin (g/L) | 37 (5) | 38 (3) | 35 (6) | 36 (5) |
| Anaerobic threshold  (ml/kg/min) | 13 (3) | 17 (7) | 15 (2) | 16 (3) |
| VO_2_ max (ml/kg/min) | 13 (4) | 17 (7) | 15 (2) | 16 (3) |
| VE/VCO_2_ (l/l) | 33 (7) | 31 (3) | 32 (5) | 31 (4) |
| V-POSSUM | 20 (4) | 19 (4) | 17 (2) | 18 (3) |
| ACEI | 15 (71) | 12 (50) | 5 (38) | 5 (45) |
| Statin | 17(81) | 16 (67) | 9 (69) | 9 (82) |
| Beta-blocker | 7 (33) | 7 (29) | 5 (38) | 4 (36) |
| Hypertension | 16 (76%) | 16 (67%) | 10 (77%) | 9 (80%) |
| Ischaemic heart disease | 10 (48%) | 5 (21%) | 3 (23%) | 4 (36%) |
| Cerebrovascular disease | 5 (24%) | 6 (25%) | 1 (8%) | 1 (9%) |
| Congestive cardiac failure | 5 (24%) | 1 (4%) | 0 (0%) | 0 (0%) |
| Predicted complex EVAR | 10 (48%) | 1 (4%) | - | - |

**Table 3b: Clinical Outcomes by operative procedure**

Legend:

Data are shown as n (%) and cover the period from surgery to 6 months.

*Wound Infection, buttock or groin pain, pyrexia of unknown origin, graft problems.

AKIN refers to Acute Kidney Injury as classified by Acute Kidney Injury Network [26].

|  | **EVAR (n=45)** | | **OPEN (n=24)** | |
| --- | --- | --- | --- | --- |
|  | **Randomised allocation** | | **Randomised allocation** | |
|  | **RIC (n=21)** | **SHAM (n=24)** | **RIC (n=13)** | **SHAM (n=11)** |
| Acute kidney injury |  |  |  |  |
| AKIN 1 | 5 (17%) | 1 (24%) | 4 (31%) | 3 (27%) |
| AKIN 2 | 4 (4%) | 5 (19%) | 3 (23%) | 2 (18%) |
| AKIN 3 | 0 (0%) | 0 (0%) | 0 (0%) | 2 (18%) |
| Myocardial Infarction | 3 (14%) | 0 (0%) | 2 (15%) | 2 (18%) |
| New post-op ECG changes | 5 (24%) | 1 (4%) | 3 (23%) | 5 (45%) |
| New arrhythmia | 4 (19%) | 1 (4%) | 3 (23%) | 4 (36%) |
| Troponin T>14ng/L | 10 (48%) | 5 (21%) | 6 (45%) | 5 (45%) |
| Other post-operative issues* | 7 (33%) | 7 (29%) | 6 (46%) | 5 (45%) |
| Death | 0 (0%) | 1 (4%) | 0 (0%) | 2 (18%) |
